# Ten-Year Changes in Bloodstream Infection With *Acinetobacter Baumannii* Complex in Intensive Care Units in Eastern China: A Retrospective Cohort Study

**DOI:** 10.3389/fmed.2021.715213

**Published:** 2021-08-05

**Authors:** Xiao Meng, Jintao Fu, Yue Zheng, Weidong Qin, Hongna Yang, Dongming Cao, Haining Lu, Lu Zhang, Zhiguo Du, Jiaojiao Pang, Wei Li, Haipeng Guo, Juan Du, Chen Li, Dawei Wu, Hao Wang

**Affiliations:** ^1^The Key Laboratory of Cardiovascular Remodeling and Function Research, Chinese Ministry of Education, Chinese National Health Commission and Chinese Academy of Medical Sciences, The State and Shandong Province Joint Key Laboratory of Translational Cardiovascular Medicine, Department of Cardiology, Qilu Hospital, Cheeloo College of Medicine, Shandong University, Jinan, China; ^2^Department of Critical Care Medicine, Yanzhou Branch of Affiliated Hospital of Jining Medical University, Jining, China; ^3^Shandong Provincial Clinical Research Center for Emergency and Critical Care Medicine, Institute of Emergency and Critical Care Medicine of Shandong University, Chest Pain Center, Qilu Hospital of Shandong University, Jinan, China; ^4^Department of Emergency Medicine, Qilu Hospital of Shandong University, Jinan, China; ^5^Key Laboratory of Emergency and Critical Care Medicine of Shandong Province, Key Laboratory of Cardiopulmonary-Cerebral Resuscitation Research of Shandong Province, Shandong Provincial Engineering Laboratory for Emergency and Critical Care Medicine, Qilu Hospital of Shandong University, Jinan, China; ^6^Department of Critical Care Medicine, Qilu Hospital of Shandong University, Jinan, China; ^7^Department of Critical Care Medicine, Shandong Province Hospital Affiliated to Shandong First Medical University, Shandong First Medical University, Jinan, China; ^8^Department of Critical Care Medicine, Liaocheng People's Hospital Affiliated to Shandong First Medical University, Liaocheng, China; ^9^Department of Critical Care Medicine, Qilu Hospital of Shandong University, Qingdao, China; ^10^Department of Critical Care Medicine, The Second Hospital of Shandong University, Jinan, China; ^11^Department of Critical Care Medicine, Jiaxiang People's Hosptial, Jining, China; ^12^Department of Clinical Laboratory, Qilu Hospital of Shandong University, Jinan, China; ^13^Department of Pharmacology, School of Basic Medical Sciences, Shandong University, Jinan, China

**Keywords:** *Acinetobacter baumannii* complex, bloodstream infection, antibiotic resistance, prognosis, eastern China

## Abstract

**Background:** There is little evidence on the changing prevalence, microbiological profile, and outcome of nosocomial *Acinetobacter baumannii* complex (ABC)-caused bloodstream infection (ABCBSI) specified in intensive care units (ICUs) in long-term studies, especially in China.

**Objective:** We aimed to investigate changes in incidence, antibiotic resistance, therapy, and prognosis of ABCBSI in ICUs in eastern China during 2009–2018.

**Methods:** A multicenter retrospective cohort study was conducted, and microbiological and clinical data for patients with ABCBSI acquired in nine adult ICUs in eastern China from 2009 to 2018.

**Results:** A total of 202 cases were enrolled. For the years 2009–2010, 2011–2012, 2013–2014, 2015–2016, and 2017–2018, the incidence of ABCBSI increased significantly, as did the percentage of pan-drug-resistant isolates and resistant rates to most of antimicrobial agents; the percentage of drug-sensitive isolates decreased (all *P* < 0.05). The frequency of treatment with carbapenems and tigecycline increased, and that of cephalosporins decreased. Compared with those in the first years (2009–2012), ABCBSI patients in the lattermost years (2017–2018) were less often treated with appropriate empirical therapy, more often underwent pneumonia-related ABCBSI and mechanical ventilation support, and had higher 28-day mortality rates. Multivariate Cox regression indicated that increase in the degree of ABC antibiotics resistance, pneumonia-related ABCBSI, and septic shock were risk factors of 28-day mortality and associated with significant lower survival days.

**Conclusions:** The past decade has witnessed a marked increase in the incidence of ABCBSI and in antibiotic resistance, with increasing pneumonia-related infections and worrisome mortality in ICUs in China. Controlling increasing resistance and preventing nosocomial pneumonia may play important roles in combatting these infections.

## Introduction

Bloodstream infections (BSIs) are frequent and life-threatening in hospitals (1). Patients in intensive care units (ICUs) are particularly pre-disposed to BSIs (2), with a prevalence of ~15% (3). The *Acinetobacter baumannii* complex (ABC) has great potential for nosocomial spread (4). It has become an important cause of BSI among patients in ICUs (1, 5), and ABC-caused BSI (ABCBSI) is associated with high mortality (5–7).

Until recently, we had little evidence on the long-term changes in prevalence, microbiological profile, therapy, and outcome of ABCBSI in ICUs over the years. Investigating these dynamic changes has become extremely urgent, especially in China. Although the epidemiology of ABC infection and the antimicrobial susceptibility profiles of ABC isolates vary by regions, years, and even wards all over the world (8–12), the prevalence of ABC nosocomial infection seems extremely challenging in ICUs in China (13–15). Recent reports from Chinese ICUs found that ABC accounted for nearly one-quarter of the ICU-acquired infections (13, 14). The China Antimicrobial Surveillance Network (CHINET) surveillance system also found a large fluctuation in resistance of ABC strains over time in Chinese hospitals (16), which indicates that antibiotic implementation may also have significantly changed over time.

In this study, our aim was to investigate the changes in incidence, antibiotic resistance, antimicrobial treatment, and prognosis of ABCBSI in nine eastern Chinese ICUs over the past decade.

## Methods

### Study Design and Patient Selection

This was a multicenter retrospective cohort study of ABCBSI in nine mixed adult ICUs in eastern China: Qilu Hospital of Shandong University (two ICUs), Second Hospital of Shandong University, Qingdao Branch of Qilu Hospital, Liaocheng People's Hospital affiliated with Taishan Medical College, Zaozhuang Hospital, Jiaxiang People's Hospital, Shenxian People's Hospital, and Chengwu People's Hospital. We included adult patients (age ≥ 18 years) diagnosed with ICU-acquired ABCBSI from January 2009 to December 2018. ABCBSI was determined according to the surveillance definition of the US Centers for Disease Control and Prevention (17). Patients were included only if blood cultures were obtained 2 days or more after ICU admission (denoting ICU nosocomial infection). The sources of ABCBSI were assessed by study investigators according to clinical symptoms, signs, imaging data, surgical findings, and microbiological evidence. Microbiological evidence refers to the isolation from the source of the same organism that was isolated in blood culture (i.e., *A. baumannii*) (18). For patients with multiple ABCBSI episodes, only the first episode was included. Patients were excluded if they (1) had a length of ICU stay <72 h; (2) had an incomplete medical history or were lost to follow-up; (3) had their strain isolated within 48 h after admission; or (4) had previously been infected with ABC in another ward or hospital before ICU admission. The patients with coinfection by other pathogens at other sites were not excluded. The study was approved by the ethics committees of each hospital.

### Microbiological Studies

Blood cultures were processed at the participating hospitals by the BACTEC system (Becton Dickinson, Franklin Lakes, NJ, USA) or BacT/ALERT 3D system (bioMérieux, Marcy-l'Étoile, France). The blood culture bottles were incubated in the above two blood culture systems until a positive alert was gotten or for a maximum of 5 days. Two or three drops of positive blood culture broth were streaked onto the 5% sheep blood agar plate or MacConkey agar plate, respectively, and all the plates were incubated at 5% CO_2_ and 35°C. ABC isolates were Gram-negative, non-fermentative, and oxidase-negative coccobacillus using the Gram stain and manual biochemical tests, and they were identified using the VITEK-2 compact system with the GN identification card (bioMérieux, Marcy-l'Étoile, France) or MicroScan WalkAway plus system with the NC50 card (Siemens Healthcare Diagnostics, West Sacramento, CA, USA) according to the manufacturers' manual. The antibiotic susceptibility testing (AST) of ABC isolates was performed on the VITEK-2 compact system with the AST-GN16 card or MicroScan WalkAway plus System with the NC50 card. The strains of *Escherichia coli* ATCC 25922 and *Pseudomonas aeruginosa* ATCC 27853 were used as quality controls to ensure the credibility of identification and AST results of ABC isolates.

Multidrug-resistant (MDR), extensively drug-resistant (XDR), and pan-drug-resistant (PDR) isolates were defined according to international expert proposals (19). For *A. baumannii*, antimicrobial categories and agents used to define drug resistance include aminoglycosides (gentamicin, tobramycin, amikacin, and netilmicin), antipseudomonal carbapenems (imipenem, meropenem, and doripenem), antipseudomonal fluoroquinolones (ciprofloxacin and levofloxacin), antipseudomonal penicillins + β-lactamase inhibitors (piperacillin–tazobactam and ticarcillin–clavulanic acid), extended-spectrum cephalosporins (cefotaxime, ceftriaxone, ceftazidime, and cefepime), folate pathway inhibitors (trimethoprim–sulfamethoxazole), penicillins + β-lactamase inhibitors (ampicillin–sulbactam), and tetracyclines (tetracycline, doxycycline, and minocycline). MDR was defined as non-susceptible to at least one agent in three or more antimicrobial categories. XDR was defined as non-susceptible to at least one agent in all but two or fewer antimicrobial categories, and PDR was non-susceptible to all agents listed. The remaining isolates were defined as sensitive isolates. Because polymyxin has not been available in mainland China since 2018, this agent was not tested in the laboratory and was not included in the antimicrobial agents list (19). To better define the resistance patterns and resistance degree of each ABC isolate, we counted the number of sensitive, MDR (excluding XDR), XDR (excluding PDR), and PDR isolates.

### Data Collection

Trained study team members collected data by chart review. Demographic and microbiological data, comorbidities, precipitating factors, laboratory data, concurrent infections with other pathogens in the bloodstream, antibacterial agent treatment, and outcome were recorded on standardized case report forms. At the onset of ABCBSI (within 24 h after collection of the first ABC-positive blood sample), laboratory data were collected, and the severity of the initial presentation of ABCBSI was assessed by APACHE II scores. The definition of concurrent infection with (an)other pathogen(s) in the bloodstream was that one or more other pathogens were isolated from cultures of blood samples obtained within 48 h of collection of the first ABC-positive blood sample, irrespective of whether the isolate came from the same or a different blood culture bottle (20). APACHE II scores were calculated according to a previous report. They use 12 acute physiological variables, age, and chronic health status (21). The primary outcome was survival at 28 days after ABCBSI onset. Patients discharged from the hospital were followed up by the medical electronic system or by telephone to determine their survival status. We defined empirical therapy as being an antimicrobial regimen administered within 24 h of extraction of a blood sample and before susceptibility was known; a therapy that was continued or commenced on the day that antibiogram results were reported was considered definitive (antibiogram-directed therapy) (22). Appropriate empirical treatment was defined as prescription of one or more antimicrobial active against the isolate of ABC, in accordance with the standard antimicrobial susceptibility study, in the first 24 h from the onset of the bacteremia and before its diagnosis, with an approved route and dosage appropriate for end organ function. Appropriate definitive treatment was antibiotic active against the isolate of ABC within 24 h after obtaining the antibiogram, with an approved route and dosage appropriate for end organ function (2). Adequate source control was defined as removal of any pre-existing devices thought to be the source of BSI or documented interventions using appropriate decompression, debridement, drainage, and other surgical procedures to control the infection source within 48 h of the onset of BSI (23). Adequacy of source control was assessed independently by a multidisciplinary panel of experts composed of one infectious disease specialists, one intensivist, and a surgeon (all with more than 10 years of experience). Septic shock was diagnosed according to the sepsis-3 definition (24).

The variables included in the 28-day mortality analysis were age, sex, resistance patterns of AB isolates (sensitive, MDR, XDR, and PDR), Charlson Comorbidity Index (25), source of ABCBSI, days from ICU admission to ABCBSI, polymicrobial BSI (26), the severity of infection according to APACHE II scores (27), the presence of septic shock (at ABCBSI diagnosis) (28), appropriate antimicrobial therapy (29), combined antimicrobial treatment (28), invasive procedures (including invasive ventilation and renal replacement therapy) (27, 29), and inadequate source control (30).

### Statistical Analyses

SPSS 16.0 (SPSS Inc., IL, USA) was used for data analysis. Categorical data are shown as number (%) and continuous variables as mean ± SD. Chi-square or Fisher's exact-test (two-tailed) was used to compare categorical variables. Unpaired Student's *t*-test or one-way ANOVA was used to compare continuous variables. Cox proportional hazards regression estimated hazard ratios (HRs) and 95% confidence intervals (CIs) of risk factors for mortality and included all the above potential confounder variables. We set the ordered resistance patterns as a one-degree-of-freedom linear term in the Cox regression model. *P* < 0.05 was considered statistically significant.

## Results

### Patient Demographics and the Incidence of ABCBSI by Years

Two hundred and twenty-eight patients presented ABCBSI between 2009 and 2018; we excluded 26 cases: length of stay <72 h (*n* = 4); incomplete medical history or lost to follow-up (*n* = 3); and ABC strain isolated within 48 h after admission or previous infection with ABC in another ward or hospital before ICU admission (*n* = 19). The mean age of enrolled participants (*n* = 202) was 59.5 ± 16.9 years; 65.3% were male. The mean total length of ICU stay (mean ICU stay) was 20.6 ± 14.3 days, and the overall 28-day mortality was 34.2%. For the years 2009–2010, 2011–2012, 2013–2014, 2015–2016, and 2017–2018, a significantly increased incidence of ABC infection was observed (99, 285, 498, 672, and 926 per 1,000,000 ICU population, respectively, *P* < 0.01). The three infection sources of ABCBSI were the lung (53.5%), abdomen and pelvis (11.9%), and catheter-related BSI (6.4%).

### Microbiology of ABC Isolates

**Figure 1:**
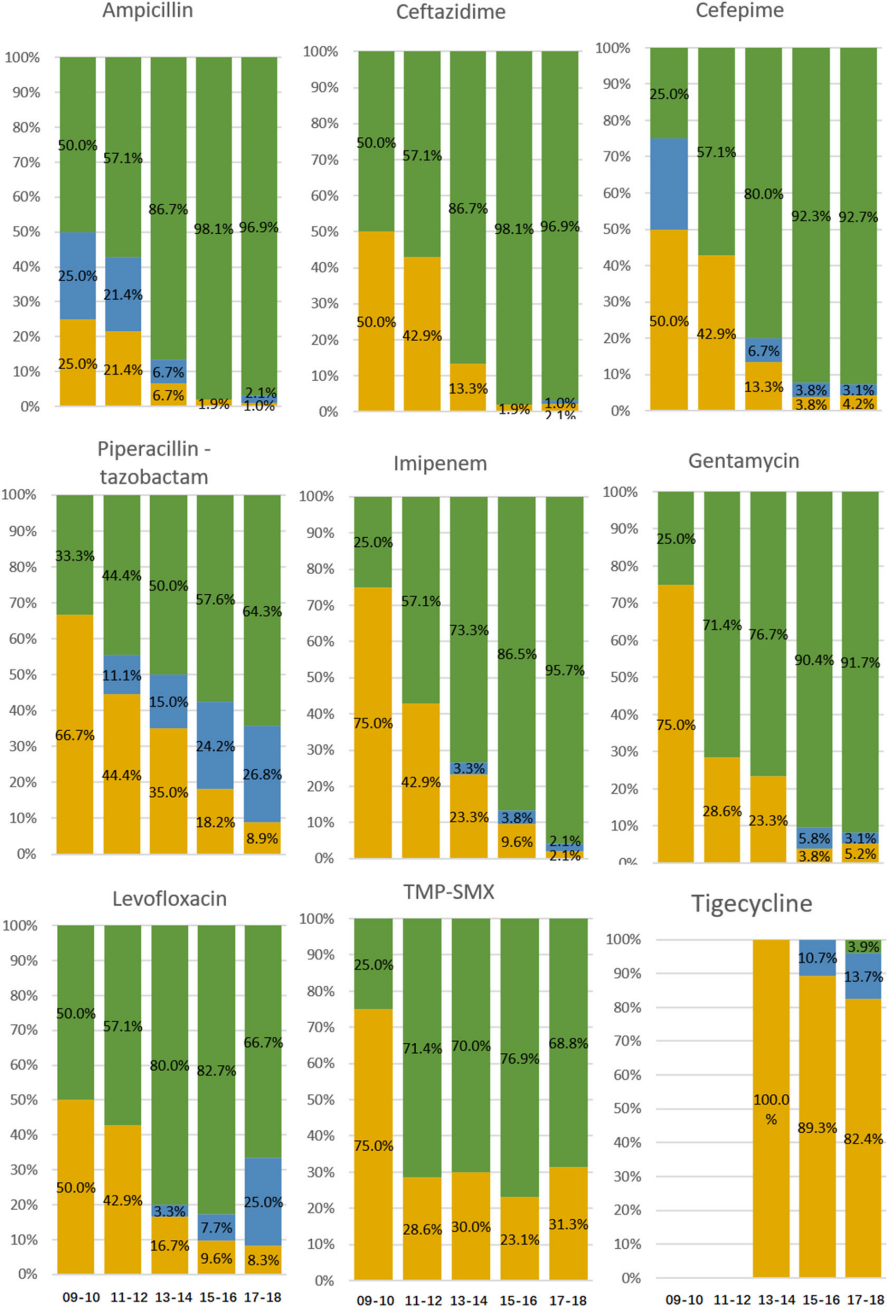
Resistance to antibacterial agents of ABC isolates from patients diagnosed with ICU-acquired ABCBSI. Data are presented as *percentage*. Green bars, resistant isolates; blue bars, intermediate isolates; yellow bars, sensitive isolates.

For the years 2009–2010, 2011–2012, 2013–2014, 2015–2016, and 2017–2018, the PDR strains increased, and sensitive strains decreased significantly (all *P* < 0.05), but the proportions of MDR (excluding XDR) and XDR (excluding PDR) strains did not significantly change (Table 1). The rates of sensitivity to ampicillin, ceftazidime, cefepime, imipenem, gentamycin, levofloxacin, and piperacillin–tazobactam decreased significantly (all *P* < 0.05), with no significant change in rates of intermediate resistance to ceftazidime, cefepime, imipenem, gentamycin, levofloxacin, trimethoprim/sulfamethoxazole, or piperacillin–tazobactam except ampicillin (Figure 1). No ABC isolates were tested for tigecycline resistance until 2012, and for the years 2013–2014, 2015–2016, and 2017–2018, the rates of sensitivity to tigecycline decreased, although not significantly (*P* > 0.05).

**Table 1:** Demographics and clinical data for patients with ABCBSI in different years.

**Characteristics**	**Total (***n*** = 202)**	**2009–2010 (***n*** = 4)**	**2011–2012 (***n*** = 14)**	**2013–2014 (***n*** = 32)**	**2015–2016 (***n*** = 54)**	**2017–2018 (***n*** = 98)**	***P*** **-value**
Age (years)	59.5 ± 16.9	63.8 ± 9.5	60.0 ± 14.6	60.3 ± 18.3	60.3 ± 16.2	58.0 ± 17.5	0.944
Male sex	132 (65.3%)	3 (75.0%)	8 (57.1%)	22 (68.8%)	36 (66.7%)	63 (64.3%)	0.933
Charlson index	2.7 ± 1.6	3.3 ± 2.1	2.9 ± 1.8	2.7 ± 1.4	2.7 ± 1.8	2.6 ± 1.5	0.925
**Comorbidity diseases**							
Type II diabetes mellitus	56 (27.7%)	1 (25.0%)	5 (35.7%)	9 (28.1%)	15 (27.8%)	26 (26.5%)	0.970
Solid tumor	37 (18.3%)	1 (25.0%)	4 (28.6%)	6 (18.8%)	9 (16.7%)	17 (17.3%)	0.867
Hematologic malignancy	7 (3.5%)	0 (0.0%)	0 (0.0%)	2 (6.3%)	2 (3.7%)	3 (3.1%)	0.836
Chronic renal insufficiency	23 (11.4%)	0 (0.0%)	2 (14.3%)	3 (9.4%)	6 (11.1%)	12 (12.2%)	0.934
**Resistance patterns of ABC isolates**							
1 (Sensitive isolates)	29 (14.4%)	2 (50.0%)	6 (42.9%)	7 (21.8%)	6 (11.1%)	8 (8.1%)	**0.001**
2 (MDR, excluding XDR)	12 (5.9%)	1 (25.0%)	2 (14.3%)	4 (12.5%)	2 (3.7%)	3 (3.1%)	0.068
3 (XDR, excluding PDR)	71 (35.1%)	1 (25.0%)	6 (42.9%)	10 (31.3%)	17 (31.5%)	37 (37.8%)	0.849
4 (PDR)	90 (44.6%)	0 (0.0%)	0 (0.0%)	11 (34.4%)	28 (51.9%)	51 (52.1%)	**0.001**
**The source of BSI**							
Lung	109 (53.9%)	1 (25.0%)	5 (35.7%)	15 (46.9%)	30 (55.6%)	58 (59.2%)	0.287
Intra-abdomen	25 (12.4%)	1 (25.0%)	3 (21.4%)	4 (12.5%)	7 (13.0%)	10 (10.2%)	0.719
Catheter-related BSI	13 (6.4%)	1 (25.0%)	2 (14.3%)	2 (6.3%)	3 (5.6%)	5 (5.1%)	0.395
Skin and soft tissue	11 (5.4%)	0 (0.0%)	1 (7.1%)	3 (9.4%)	3 (5.6%)	4 (4.1%)	0.805
Urinary tract	6 (3.0%)	0 (0.0%)	1 (11.1%)	1 (3.1%)	1 (1.9%)	3 (3.1%)	0.884
Others	14 (6.9%)	0 (0.0%)	1 (7.1%)	2 (6.3%)	5 (9.3%)	6 (6.1%)	0.928
Primary	24 (11.9%)	1 (25.0%)	1 (7.1%)	5 (14.6%)	5 (9.3%)	12 (12.2%)	0.781
Appropriate empirical therapy	61 (30.1%)	3 (75.0%)	6 (42.9%)	10 (31.3%)	16 (29.6%)	26 (26.5%)	0.203
Appropriate antibiogram-directed therapy	107 (53.0%)	3 (75.0%)	9 (64.3%)	18 (56.3%)	27 (50.0%)	50 (51.0%)	0.740
Inadequate source control	40 (19.8%)	1 (25.0%)	2 (14.3%)	6 (18.8%)	11 (20.3%)	20 (20.4%)	0.983
Polymicrobial BSI	32 (15.8%)	0 (0.0%)	2 (14.3%)	5 (15.6%)	9 (16.7%)	16 (16.3%)	0.935
Septic shock	121 (60.0%)	1 (25.0%)	6 (42.9%)	17 (53.1%)	35 (64.8%)	62 (63.3%)	0.254
Use of invasive ventilation	159 (78.7%)	2 (50.0%)	7 (50.0%)	22 (68.8%)	44 (81.5%)	84 (85.7%)	**0.008**
Use of renal replacement therapy	60 (29.7%)	0 (0.0%)	4 (28.6%)	7 (21.9%)	14 (25.9%)	35 (35.7%)	0.319
APACHE II score	19.8 ± 8.0	17.3 ± 10.5	17.9 ± 7.0	17.2 ± 5.8	20.3 ± 8.5	20.6 ± 8.4	0.165
ICU days	20.6 ± 14.3	19.8 ± 6.7	20.1 ± 10.8	19.3 ± 18.3	19.1 ± 13.0	21.9 ± 14.8	0.801
28-day mortality	69 (34.2%)	0 (0.0%)	2 (14.3%)	9 (28.1%)	19 (35.2%)	39 (39.8%)	0.167

*Data are n (%) or mean ± SD. ICU, intensive care unit; APACHE, Acute Physiology and Chronic Health Evaluation; MDR, multidrug resistance; XDR, extensive drug resistance; PDR, pan-drug resistance; BSI, bloodstream infection*.

*P-values < 0.05, which are considered statistically significant*.

### Use of Antibiotics, Clinical Characteristics, and Prognosis in ABCBSI Patients

**Figure 2:**
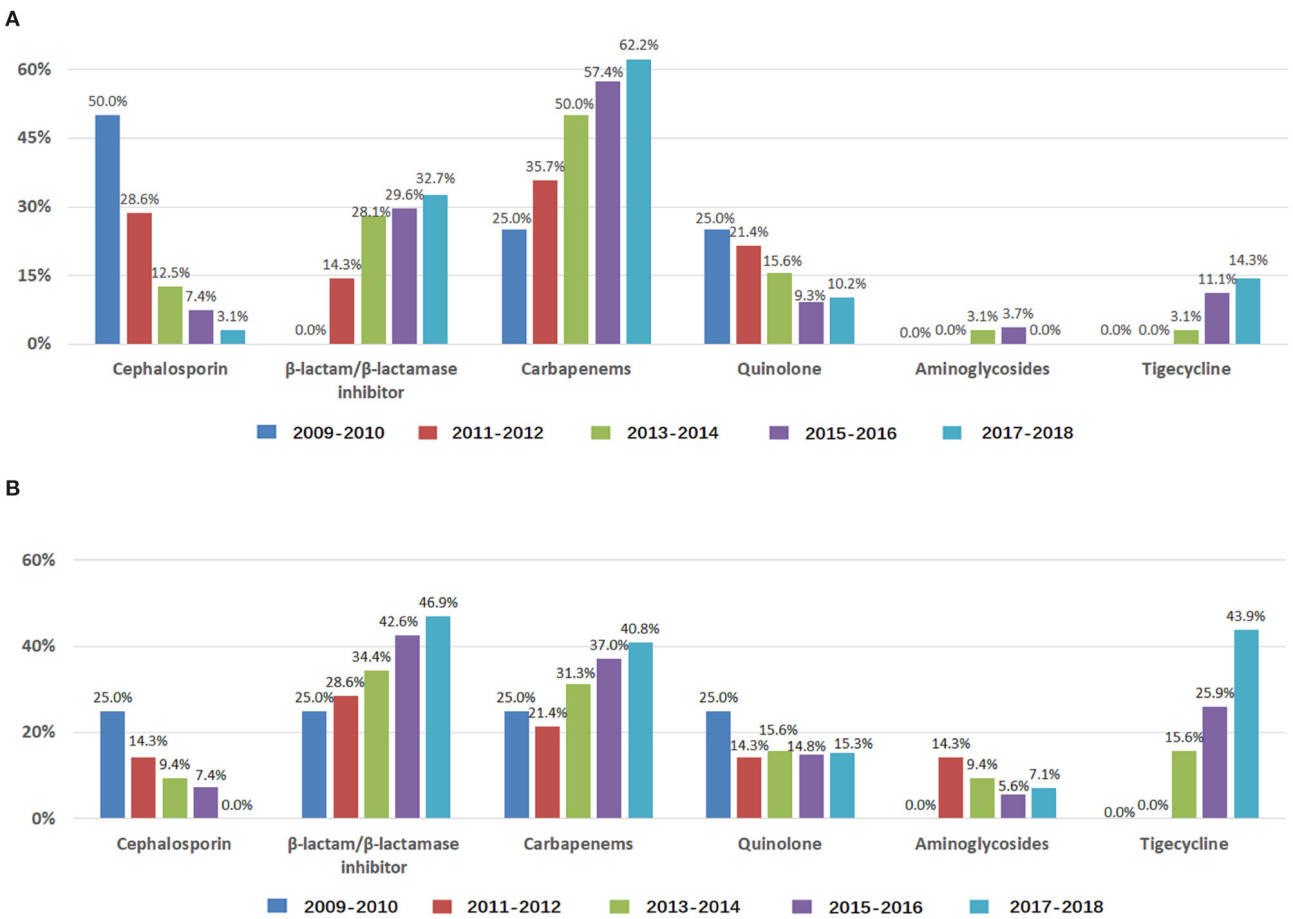
Antibiotics used to treat ABCBSI in ICUs during 2009–2018. Data are presented as *percentage*. **(A)** Use of antibiotics for empirical treatment of ABCBSI; **(B)** Use of antibiotics for antibiogram-directed treatment of ABCBSI.

For the years 2009–2010, 2011–2012, 2013–2014, 2015–2016, and 2017–2018, the frequency of empirical treatment increased with β-lactam/β-lactamase inhibitor, carbapenems, and tigecycline and decreased with cephalosporins and quinolone (Figure 2A); the frequency of antibiogram-directed treatment increased with β-lactam/β-lactamase inhibitor and tigecycline and decreased with cephalosporins (Figure 2B). ABCBSI patients did not differ in age, sex, underlying diseases, the source of ABCBSI, presence of polymicrobial BSI or septic shock, APACHE II scores, and ICU days, but the use of invasive ventilation increased (*P* < 0.05, Table 1). We found an increased 28-day mortality (0.0, 14.3, 28.1, 35.2, and 39.8%), although it was not significant (Table 1).

To better explore this long-term change, we compared the clinical characteristics and prognosis of the patients in the first years (2009–2012) and the last years (2017–2018): ABCBSI patients in the years 2017–2018 were less often treated with appropriate empirical therapy, more often underwent pneumonia-associated ABCBSI and mechanical ventilation support, and had higher 28-day mortality rates (Table 2).

**Table 2:** Comparison of indicators between years 2009–2012 and years 2017–2018 in patients with ABCBSI.

**Characteristics**	**2009–2012 (***n*** = 18)**	**2017–2018 (***n*** = 98)**	***P*** **-value**
Age (years)	60.8 ± 13.5	58.0 ± 17.5	0.509
Male sex	11 (61.1%)	63 (64.3%)	0.797
Charlson Index	3.0 ± 1.9	2.6 ± 1.5	0.632
**Comorbidity diseases**			
Type II diabetes mellitus	6 (33.3%)	26 (26.5%)	0.553
Solid tumor	5 (27.8%)	17 (17.3%)	0.299
Hematologic malignancy	0 (0.0%)	3 (3.1%)	0.956
Chronic renal insufficiency	2 (11.1%)	12 (12.2%)	0.797
**Resistance patterns of ABC isolates**			
1 (sensitive isolates)	8 (44.2%)	8 (8.1%)	**<0.001**
2 (MDR, excluding XDR)	3 (16.7%)	3 (3.1%)	0.069
3 (XDR, excluding PDR)	7 (38.9%)	37 (37.8%)	0.927
4 (PDR)	0 (0.0%)	51 (52.1%)	**<0.001**
**The source of BSI**			
Lung	6 (33.3%)	58 (59.2%)	**0.043**
Intra-abdomen	4 (22.2%)	10 (10.2%)	0.296
Catheter-related BSI	3 (16.7%)	5 (5.1%)	0.203
Skin and soft tissue	1 (5.6%)	4 (4.1%)	0.728
Urinary tract	1 (5.6%)	3 (3.1%)	0.865
Others	1 (5.6%)	6 (6.1%)	0.656
Primary	2 (11.1%)	12 (12.2%)	0.797
Appropriate empirical therapy	9 (50.0%)	26 (28.6%)	**0.046**
Appropriate antibiogram-directed therapy	12 (75.0%)	50 (51.0%)	0.221
Inadequate source control	3 (16.7%)	20 (20.4%)	0.965
Polymicrobial BSI	2 (11.1%)	16 (16.3%)	0.835
Septic shock	7 (38.9%)	62 (63.3%)	0.053
Use of invasive ventilation	9 (50.0%)	84 (85.7%)	**<0.001**
Use of renal replacement therapy	4 (22.2%)	35 (35.7%)	0.265
APACHE II score	17.8 ± 7.5	20.6 ± 8.4	0.174
ICU days	20.0 ± 9.9	21.9 ± 14.8	0.588
28-day mortality	2 (11.1%)	39 (39.8%)	**0.019**

*Data are n (%) or mean ± SD. ICU, intensive care unit; APACHE, Acute Physiology and Chronic Health Evaluation; MDR, multidrug resistance; XDR, extensive drug resistance; PDR, pan-drug resistance; BSI, bloodstream infection*.

*P-values < 0.05, which are considered statistically significant*.

### Univariate and Multivariable Analyses of Factors Associated With 28-Day Mortality in ABCBSI Patients

As compared with survivors, non-survivors more frequently had XDR and PDR strains, less frequently had sensitive isolates, more frequently had comorbidity of diabetes mellitus and lung infection-related ABCBSI, received invasive ventilation, and presented septic shock and higher APACHE II scores (all *P* < 0.05, Table 3). In the Cox regression model, the increased resistance degree of ABC isolates (HR: 2.51, 95% CI: 1.68–3.75, *P*-value for trend <0.001), lung infection-related ABCBSI (HR: 3.83, 95% CI: 1.10–13.30), and septic shock (HR: 3.08, 95% CI: 1.55–6.14) were identified as independent risk factors for mortality (Table 3). Kaplan–Meier curves visually compared survival across these three independent risk factors (Supplementary Figure 1).

**Table 3:** Univariate and multivariate Cox regression analyses of the association between different variables and 28-day mortality.

**Characteristics**	**Non-survivors (*n* = 69)**	**Survivors (*n* = 133)**	**Univariate analysis**		**Multivariable model**	
			**HR (95% CI)**	***P*** **-value**	**HR (95% CI)**	***P*** **-value**
Age (years)	62.3 ± 16.1	58.0 ± 17.2	1.01 (0.99–1.03)	0.063	0.98 (0.98–1.12)	0.995
Male sex	41 (59.4%)	91 (68.4%)	0.74 (0.46–1.20)	0.218	0.67 (0.38–1.78)	0.161
Charlson index	3.0 ± 1.7	2.6 ± 1.5	1.14 (0.99–1.31)	0.066	0.01 (0.81–1.25)	0.949
**Comorbidity diseases**						
Type II diabetes mellitus	25 (36.2%)	31 (23.3%)	1.72 (1.06–2.82)	**0.030**	1.32 (0.70–2.48)	0.391
Solid tumor	15 (21.7%)	22 (16.5%)	1.24 (0.670–2.19)	0.466	1.95 (0.96–4.43)	0.062
Hematologic malignancy	1 (1.4%)	5 (3.8%)	0.40 (0.06–2.88)	0.362	0.25 (0.03–2.18)	0.212
Chronic renal insufficiency	12 (17.4%)	11 (8.3%)	1.79 (0.96–3.34)	0.067	1.16 (0.49–2.72)	0.738
**Resistance patterns of ABC isolates**						
1 (Sensitive isolates)	1 (1.4%)	28 (21.1%)	Reference		Reference	
2 (MDR, excluding XDR)	2 (2.9%)	10 (7.5%)	5.17 (0.47–57.03)	0.180	13.34 (1.12–159.06)	**0.040**
3 (XDR, excluding PDR)	19 (27.5%)	52 (39.1%)	9.16 (1.23–68.40)	**0.031**	17.62 (2.14–145.38)	**0.008**
4 (PDR)	47 (68.1%)	43 (32.3%)	20.43 (2.82–148.15)	**0.003**	31.60 (3.96–252.37)	**0.001**
*P*-value for trend			2.35 (1.63–3.39)	**<0.001**	2.51 (1.68–3.75)	**<0.001**
**Source of BSI**						
Lung	43 (62.3%)	61 (45.9%)	1.70 (1.04–2.77)	**0.033**	3.83 (1.10–13.30)	**0.035**
Intra-abdomen	6 (8.7%)	22 (16.5%)	0.56 (0.24–1.28)	0.169	0.46 (0.11–1.93)	0.285
Skin and soft tissue	5 (7.2%)	7 (5.3%)	1.33 (0.53–3.29)	0.545	2.33 (1.15–4.77)	0.073
Catheter-related BSI	2 (2.9%)	10 (7.5%)	0.41 (0.10–1.67)	0.213	0.73 (0.011–5.10)	0.755
Urinary tract	1 (2.2%)	4 (4.7%)	0.46 (0.06–3.29)	0.437	0.68 (0.07–6.92)	0.747
Primary	6 (13.0%)	18 (13.5%)	0.90 (0.43–1.88)	0.778	0.30 (0.08–1.20)	0.090
Days from ICU admission to ABCBSI	9.3 ± 5.3	10.4 ± 6.7	0.97 (0.93–1.01)	0.112	0.96 (0.92–1.01)	0.084
Polymicrobial BSI	10 (14.4%)	22 (16.5%)	0.78 (0.40–1.53)	0.478	1.34 (0.59–3.01)	0.485
Septic shock	57 (82.6%)	64 (48.1%)	3.88 (2.08–7.22)	**<0.001**	3.08 (1.55–6.14)	**0.001**
APACHE II score	22.4 ± 7.7	18.4 ± 7.8	1.05 (1.02–1.08)	**0.001**	1.01 (0.97–1.05)	0.546
Appropriate empirical therapy	15 (21.7%)	46 (34.6%)	0.65 (0.40–1.66)	0.059	0.51 (0.23–1.16)	0.108
Appropriate antibiogram-directed therapy	34 (47.8%)	73 (55.6%)	0.91 (0.65–1.73)	0.449	1.44 (0.71–2.90)	0.308
Combined therapy	44 (63.8%)	97 (72.9%)	0.71 (0.43–1.15)	0.163	0.68 (0.40–1.17)	0.165
Inadequate source control	18 (26.1%)	22 (16.5%)	1.57 (0.92–2.68)	0.101	1.65 (0.87–3.10)	0.124
Use of invasive ventilation	62 (89.9%)	97 (72.9%)	2.76 (1.26–6.03)	**0.011**	1.27 (0.51–3.16)	0.608
Use of renal replacement therapy	25 (36.2%)	35 (26.3%)	1.38 (0.84–2.25)	0.202	0.81 (0.41–1.59)	0.534

*Data are n (%) or mean ± SD. ABC, A. baumannii complex; APACHE, Acute Physiology and Chronic Health Evaluation; MDR, multidrug resistance; XDR, extensive drug resistance; PDR, pan-drug resistance; BSI, bloodstream infection*.

*P-values < 0.05, which are considered statistically significant*.

## Discussion

This study firstly reported a long-term change in incidence, antibiotic resistance, therapy, and prognosis of nosocomial ABCBSI in ICUs in eastern China. We found that for the years 2009–2018, the incidence of ABC infection increased significantly in the ICUs of eastern China, as did the resistance rates to most antibiotics; the percentage of PDR isolates increased, and sensitive isolates decreased. Compared with those in the years 2009–2012, ABCBSI patients in the years 2017–2018 were less often treated with appropriate empirical therapy, more often underwent pneumonia-related ABCBSI and mechanical ventilation support, and had higher 28-day mortality rates. Increased antibiotics resistance degree of ABC isolates, lung infection-related ABCBSI, and presence of septic shock were risk factors of 28-day mortality.

Although ABC is notorious for its great propensity for epidemic spread and has emerged as an important causative agent of nosocomial infections, the striking 10-fold rise in incidence of nosocomial ABCBSI with worrisome mortality in China ICUs in the past decade is surprising. The increasing incidence of ABCBSI can be interpreted from several aspects. First, the growing drug resistance of *A. baumannii* means there are progressively fewer therapeutic options and creates competitive advantages for *A. baumannii* over other pathogens. Data from the China Antimicrobial Resistance Surveillance System (CARSS) and CHINET show that as the prevalence and resistance of *A. baumannii* increased, the prevalence of *Staphylococcus aureus* and *P. aeruginosa* decreased notably (31). Second, infection control depends on active surveillance, contact isolation, healthcare worker compliance with hand hygiene, and aseptic care of vascular catheters and endotracheal tubes (32, 33). Because of human resource shortages, some nursing workers are part-time, low paid, and not well-educated. However, they participate in patient services such as feeding, turnover, metering intake, and output volume; such workers may have low compliance with hand hygiene and disinfection requirements. Third, the characteristics of patients/patient treatment changed over the period of our study. Increased use of invasive ventilation over the last decade has brought about more chance for ABC infection in the lung. Since lung infection was the most common source of BSI, the dominant role of ABC in causing ventilator-associated pneumonia may have contributed to the increasing incidence of ABCBSI (15, 34). Our results suggest the urgent need for increased attention and enhanced health policies to control ABC nosocomial infection in Chinese ICUs to decrease the spread of the organism. Control depends on active surveillance, contact isolation, healthcare worker compliance with hand hygiene, and aseptic care of vascular catheters and endotracheal tubes (32, 33).

A study from one Chinese hospital revealed that the MDR rate of ABCBSI increased significantly from 2010 to 2013 (64.71, 73.47, 75.00, and 83.05%, respectively, *P* = 0.032) (35). However, in our study, to better define the resistance degree of each ABC isolate, we counted the number of MDR (excluding XDR) isolates and did not find a significant change in its frequency between the years. Instead, the frequency of PDR isolates increased and that of sensitive isolates decreased significantly. Particularly, the rates of resistance to imipenem in Chinese ICUs continually increased (25.0, 57.1, 73.3, 86.5, and 95.7%) by years. In the year 2016, the resistance rate to imipenem was 88.5%, which is higher than that from CHINET data (68.6%) (36), mostly from general wards of Chinese hospitals.

β-Lactamase inhibitor (sulbactam and tazobactam)-based combinations have shown effective antibacterial activity for ABC strains. As shown in our study, the ABC isolates still had 35.7% intermediate and sensitive rates to piperacillin–tazobactam, so β-lactamase inhibitors could have relatively high anti-ABC activity and could be used for MDR treatment, but increasing resistance should also be considered when applied. As compared with colistin, which was unavailable until 2018, tigecycline was commercially available in 2013 in China and serves as the last defense against these persistent bacteria under most circumstances. However, an increasing resistance to tigecycline with the wide use of this agent after the year 2013 was shown and should be paid more attention. Trimethoprim/sulfamethoxazole was little used to treat ABCBSI because there was no intravenous formulation of trimethoprim/sulfamethoxazole available in mainland China; the resistance to trimethoprim/sulfamethoxazole was stable over the years in this study.

The increasing incidence of antibiotic resistance of ABC has become a worldwide challenge in healthcare facilities. A major driver of this emerging issue is the inappropriate use of antimicrobials. Mechanisms of drug resistance of ABC include expression of drug-inactivating enzymes, modifications of the target sites, increased efflux pumps, and decreased membrane permeability (37), on which the use of broad-spectrum antimicrobials exerted dramatic selective pressure. Over the years, we observed increased incidence of imipenem-resistant and pan-drug-resistant ABC, which is consistent with the increased use of carbapenem and tigecycline in Chinese ICUs. In China, ICU physicians often select carbapenems as a first-line empirical antibiotics in patients with sepsis or septic shock because of their broad-spectrum antibacterial activity, effects on bacteria that produce extended-spectrum β-lactamase, and better cost performance. Carbapenems and β-lactamase inhibitors, in combination with other agents, are still commonly used to treat CRAB infections in China (38). The extensive use of carbapenems may contribute to the growing expression of various carbapenemases, which is the hallmark of the XDR phenotype. In addition, the wide use of tigecycline in recent years is an important external factor for the overexpression of multidrug efflux pumps, which is a key molecular mechanism for the decreased susceptibility to tigecycline in *Acinetobacter* isolates (39, 40). Moreover, the excessive application of antimicrobial agents in agriculture and livestock production, especially in developing countries, also leads to increased exposure to antibiotics and worsens the situation of drug resistance (41, 42). Standardized management of antibiotics, appropriate empirical treatment, and timely de-escalation therapy may be essential for alleviating the rise of antibiotic resistance.

From epidemiological studies, the mortality rates with ABC-associated infections range from 10 to 43% in ICUs, which significantly increases the burden of ICUs (43). The overall 28-day mortality rate of ABCBSI in this study (34.2%) is comparable. However, we showed an increase in mortality (0.0, 14.3, 28.1, 35.2, and 39.8%) over the years. Studies have shown that MDR or XDR isolates were associated with longer hospitalization, increased costs, and an excess attributable mortality rate in ABCBSI patients (44, 45); therefore, they may contribute to a high rate of treatment failure led by the propensity for antimicrobial resistance (46, 47) and high frequency of biofilm forming in higher-resistance ABC (48). Necessary implementation to control the increased resistance may benefit the survival of ABCBSI patients.

In this study, lung infection-related ABCBSI was a risk factor of 28-day mortality. Although ABC can be isolated from various clinical specimens from hospitalized patients, it is commonly isolated in ICUs from intubated patients (49); thus, lower respiratory tract infections were the most common source of ABCBSI acquired in ICUs. Compared to those without pneumonia, the hospital-acquired pneumonia-related ABCBSI group were more frequently treated in ICU, had significantly higher incidence of antibiotic resistance (50), and presented with septic shock in carbapenem-resistant ABCBSI (7). Furthermore, considering nearly 80% of lung infections with ABC were hospital acquired, prevention of nosocomial ABC pneumonia may play an important role to combat these infections and decrease high mortality.

Our study has several limitations and should be interpreted with discretion. First, because molecular studies are not routinely performed and the clinical isolates are not routinely preserved in some of the participating hospitals, we did not investigate the molecular epidemiology of ABC identified by conventional biochemical methods in this study. Second, though the participants were consecutively enrolled, the clinical data were collected retrospectively, which may lead to implied bias. Third, because the study was conducted in adult ICUs in eastern China, the conclusions may not apply to different populations or different clinical situations. Additionally, patients with coinfection by other pathogens at other sites were not specifically identified and excluded, which may cause latent bias to the results.

## Conclusions

In summary, the past decade has witnessed a marked increase in incidence of ABCBSI and in antibiotic resistance, with increasing pneumonia-related infections and worrisome mortality in ICUs in eastern China. Considering that increased antibiotic resistance of ABC isolates and lung infection-related ABCBSI were risk factors for death, controlling increasing resistance of ABC and preventing nosocomial pneumonia may play important roles in combatting these infections.

## Data Availability Statement

The raw data supporting the conclusions of this article will be made available by the authors, without undue reservation.

## Ethics Statement

The studies involving human participants were reviewed and approved by the Ethics Committees of Qilu Hospital of Shandong University, Second Hospital of Shandong University, Qingdao Branch of Qilu Hospital, Liaocheng People's Hospital affiliated with Taishan Medical College, Zaozhuang Hospital, Jiaxiang People's Hospital, Shenxian People's Hospital and Chengwu People's Hospital. Written informed consent for participation was not required for this study in accordance with the national legislation and the institutional requirements.

## Author Contributions

XM contributed to manuscript writing and data analysis. JF contributed to information collection. YZ helped in manuscript preparation. WQ, HY, DC, and HL helped in data interpretation. LZ, ZD, JP, WL, HG, JD, CL, and DW participated in data collection. HW contributed to study design and manuscript writing. All authors approved the final manuscript and are responsible for the content.

## Conflict of Interest

The authors declare that the research was conducted in the absence of any commercial or financial relationships that could be construed as a potential conflict of interest.

## Publisher's Note

All claims expressed in this article are solely those of the authors and do not necessarily represent those of their affiliated organizations, or those of the publisher, the editors and the reviewers. Any product that may be evaluated in this article, or claim that may be made by its manufacturer, is not guaranteed or endorsed by the publisher.
