# Supplementary material for: Ten-Year Changes in Bloodstream Infection With Acinetobacter Baumannii Complex in Intensive Care Units in Eastern China: A Retrospective Cohort Study
Source: Front Med (Lausanne). 2021 Aug 5;8:715213. doi: 10.3389/fmed.2021.715213 (PMC8374942; doi:10.3389/fmed.2021.715213)
Supplement: Supplementary file 1 [file Image_1.PDF]

Supplementary Material

A

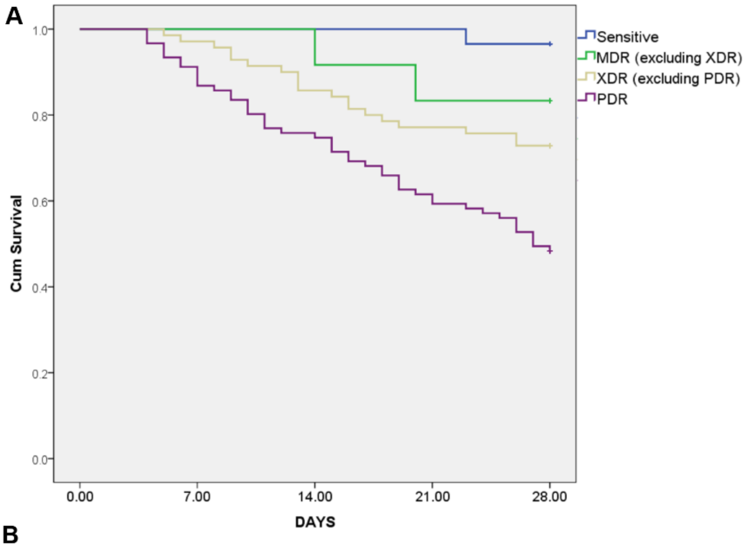

B

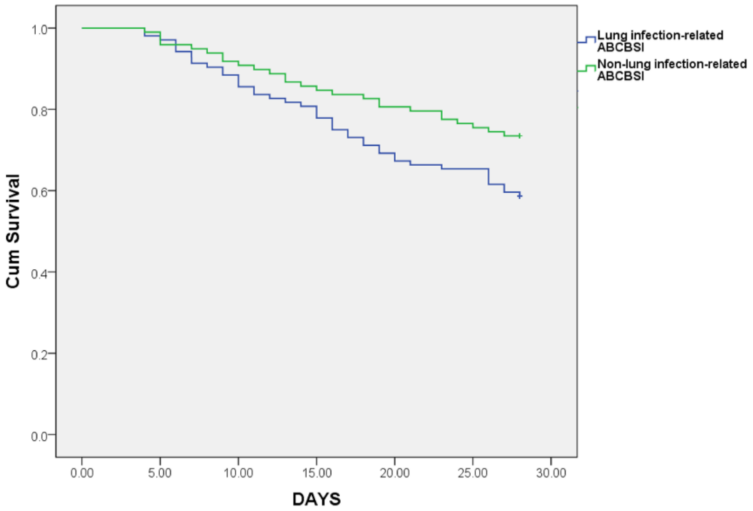

C

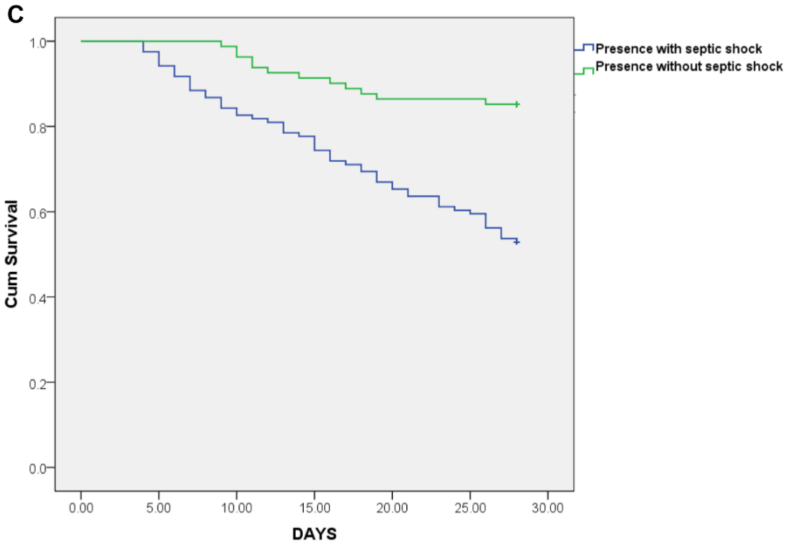

Fig. S1 Kaplan-Meier survival analysis of patients diagnosed with ICU acquired *A. baumannii* complex bloodstream infection (ABCBSI)

The survival curves were stratified by resistant degree of *A. baumannii* complex.

(A) lung infection and non-lung infection-related ABCBSI; (B) presence with and without septic shock; (C) all *P* values of log-rank tests < 0.05. MDR, multidrug-resistance; XDR, extensively drug-resistance; PDR, pandrug resistance.
